# Field trial of a probiotic bacteria to protect bats from white-nose syndrome

**DOI:** 10.1038/s41598-019-45453-z

**Published:** 2019-06-24

**Authors:** Joseph R. Hoyt, Kate E. Langwig, J. Paul White, Heather M. Kaarakka, Jennifer A. Redell, Katy L. Parise, Winifred F. Frick, Jeffrey T. Foster, A. Marm Kilpatrick

**Affiliations:** 1 Department Ecology and Evolutionary Biology, University of California, Santa Cruz, 95064 USA; 2 Department Biological Sciences, Virginia Tech, Blacksburg, VA 24061 USA; 3 Wisconsin Department of Natural Resources, Bureau of Natural Heritage Conservation, Madison, WI 53707 USA; 4 Department of Molecular, Cellular & Biomedical Science, University of New Hampshire, Durham, NH 03824 USA; 5 Present Address: Center for Microbial Genetics and Genomics, Northern Arizona University, Flagstaff, AZ 86011 USA; 6 Bat Conservation International, Austin, Texas 78716 USA

**Keywords:** Ecological epidemiology, Conservation biology, Fungal infection

## Abstract

Tools for reducing wildlife disease impacts are needed to conserve biodiversity. White-nose syndrome (WNS), caused by the fungus *Pseudogymnoascus destructans*, has caused widespread declines in North American bat populations and threatens several species with extinction. Few tools exist for managers to reduce WNS impacts. We tested the efficacy of a probiotic bacterium, *Pseudomonas fluorescens*, to reduce impacts of WNS in two simultaneous experiments with caged and free-flying *Myotis lucifugus* bats at a mine in Wisconsin, USA. In the cage experiment there was no difference in survival between control and *P*. *fluorescens*-treated bats. However, body mass, not infection intensity, predicted mortality, suggesting that within-cage disturbance influenced the cage experiment. In the free-flying experiment, where bats were able to avoid conspecific disturbance, infection intensity predicted the date of emergence from the mine. In this experiment treatment with *P*. *fluorescens* increased apparent overwinter survival five-fold compared to the control group (from 8.4% to 46.2%) by delaying emergence of bats from the site by approximately 32 days. These results suggest that treatment of bats with *P*. *fluorescens* may substantially reduce WNS mortality, and, if used in combination with other interventions, could stop population declines.

## Introduction

White-nose syndrome (WNS), caused by the introduced fungal pathogen, *Pseudogymnoascus destructans*, has caused widespread declines in bat populations throughout eastern and central North America and threatens several species with extinction^1–6^. Three species (*Myotis lucifugus*, *Myotis sodalis*, and *Perimyotis subflavus*) have declined by 70–90% across multiple states, and a fourth species, *Myotis septentrionalis*, has been extirpated from most sites within three years of WNS detection^2,3,7^, in part, due to highly connected bat communities^8^. Although a few populations of *M*. *lucifugus* appear to be persisting at 10–25% of pre-WNS colony sizes, most colonies of this species have declined by >90%^7,9^. Several previously common species of hibernating bats are now relatively rare across large regions of the northeast USA^2,7,10^. Management interventions to reduce the impact of WNS on bat populations are needed to prevent further declines and restore bat populations.

Over the past seven years, several treatments for WNS have been explored and are in various stages of development. Potential treatments to enable bats to survive hibernation have included volatile compounds released by bacteria, vaccination, chemical anti-fungals, and probiotic microbes (Table S1). However, the results of most lab and field trial studies have not been published, and there are currently no published reports of effective treatments from field trials (Table S1). Thus, at present, there are few tools for managers to reduce the impacts of WNS, and developing control options to reduce the severity of this disease among bats is a high priority^11,12^.

Our goal was to determine the efficacy of a probiotic bacterial treatment, *Pseudomonas fluorescens*, in reducing WNS mortality in a field setting. *Pseudomonas fluorescens* is a ubiquitous bacterial species complex that is used as a fungal biocontrol agent in agriculture, and has been tested as a treatment for chytridiomycosis in amphibians^13–15^. A previous study on multiple isolates of *P*. *fluorescens* isolated from different species of bats showed a range of anti-*P*. *destructans* properties *in vitro*^16^. One strain, isolated from a hibernating *Eptesicus fuscus* in Virginia, reduced the number of lesions and increased survival of little brown bats when applied at the time of infection in a laboratory *in vivo* trial^17^.

We performed a field trial with two simultaneous experiments to balance the strengths and weaknesses of each approach. In the free-flying experiment, we treated bats and attached a passive integrated transponder (PIT) tag to each bat to determine the date it emerged from the site. This experiment allowed bats to move freely throughout the site and roost wherever they chose. However, there was additional uncertainty in determining the survival of free flying bats. Bats might die at the site and be eaten by predators, escape from the site by an unknown exit and not be detected by the PIT tag receiver, or move to another site midwinter and survive at that site. To balance these unknowns, we also performed an experiment with bats in cages. Placing bats in cages, as has been done in most *in vivo* experiments to date^1,17–19^, prevents bats from leaving the site and provides certainty about the survival of each bat. However, caging bats alters their behavior (e.g. bats cannot select their roosting location and microclimate, and bats in the same cage may be disturbed when other bats arouse from hibernation^20^), and placing all bats in a treatment within a single cage results in pseudo-replication.

## Results

**Figure 1:**
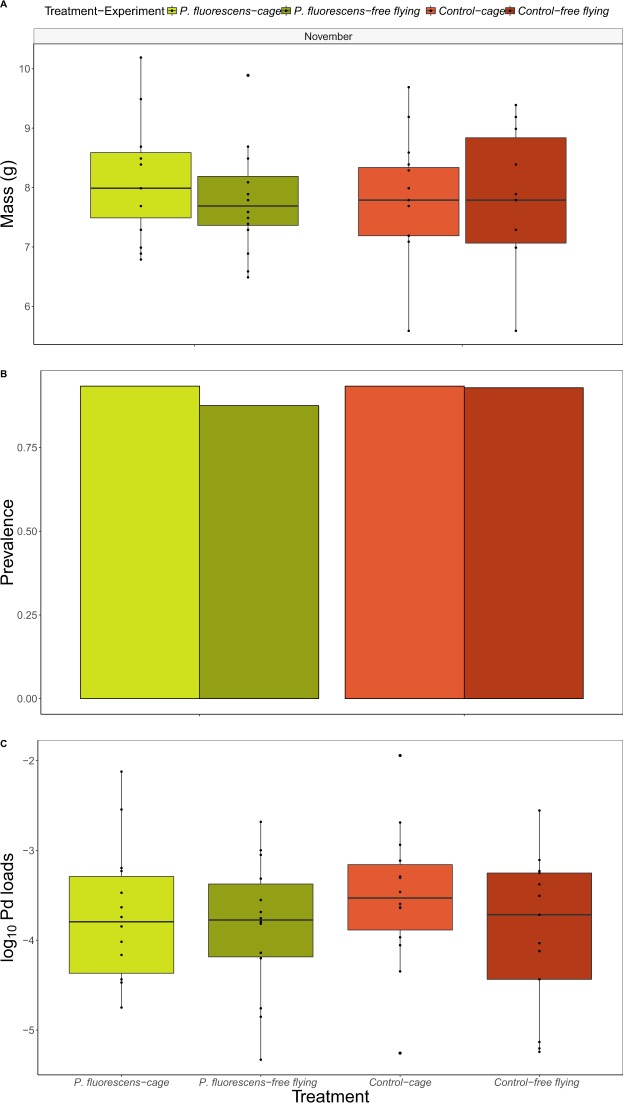
Bat body mass (**A**), *P*. *destructans* prevalence (**B**), and fungal loads (**C**) of *M*. *lucifugus* in November in two treatment groups (control (CO), and *P*. *fluorescens* (PF). There were 14–16 bats in each of the four treatment-experiment groups. There were no significant differences among treatment groups in fungal loads, prevalence or body mass in November (likelihood ratio-tests for treatment effect in linear models of mass, generalized linear models with a binomial distribution for prevalence, and linear models for log-transformed fungal loads: all P-values > 0.57).

On the day of treatment, *P*. *destructans* infection prevalence, fungal loads and weights of bats were very similar among control and treatment groups in both experiments, suggesting that randomization of bats among treatment groups was successful (Fig. 1). In addition, infection prevalence and fungal loads in November were very similar to loads observed on *M*. *lucifugus* at other sites where the fungus has been present for at least one previous winter^21,22^.

### Free-flying experiment

**Figure 2:**
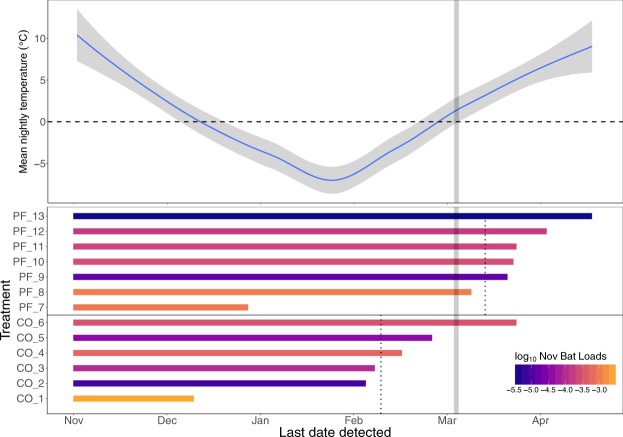
External air temperature, emergence date and fungal loads of bats in the free-flying bat experiment. The top panel shows air temperature measured at the site entrance. In the lower panel, line color indicates the log_10_ *P*. *destructans* fungal load (in nanograms) measured on bats in November, with darker colors showing lower (more negative) fungal loads. Lines end on the last date that each bat was detected by the PIT tag reader, and dotted vertical lines show the mean emergence date for each treatment group, indicated on the Y-axis (Pf – *P*. *fluorescens*, Co – Control). The gray vertical bar shows the date after which emerging bats were assumed to have survived.

**Figure 3:**
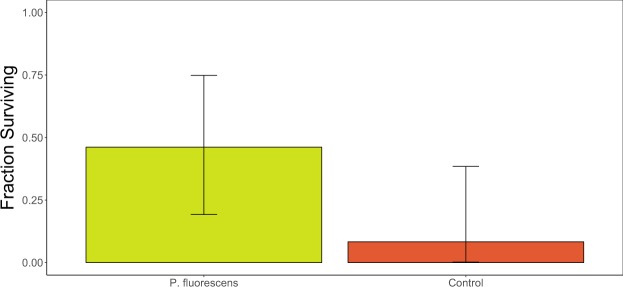
Apparent overwinter survival of bats in the free-flying experiment. Columns show the fraction of bats in each treatment group that were known to be alive and detected by the PIT tag reader on or after March 8 (with binomial 95% CI; sample sizes for each group, left to right, were 13, 12; the remaining 7 bats had lost their PIT tag; see Methods). Survival among treatments was significantly different (*P*. *fluorescens* – treated bats had higher apparent survival than the control group (Fisher’s exact test: one-tailed P = 0.046; logistic regression coef. = 2.24 ± 1.18, z = 1.896, one-tailed P = 0.029).

Of the 44 bats we treated, only one bat (from the control group) appeared to have left the site due to the disturbance of being handled/treated (it was detected by the PIT tag reader on the day of treatment November 20, 2015 and never again). We detected 13 of 29 bats in the control and *P*. *fluorescens* treatment groups on the PIT tag reader between December 9, 2015 and April 17, 2016. Six of seven *P*. *fluorescens*-treated bats, and one of six control bats left the site on or after the assumed overwinter survival date of March 8, 2016; the rest emerged earlier and we assumed they died (Fig. 2). We also found two additional bat carcasses from the control group inside the site. The fraction of bats known to be alive and detected by the PIT tag reader after March 8 (apparent overwinter survival) was 46.2% (6/13) for *P*. *fluorescens*-treated bats, which was significantly higher than 8.5% (1/12) for control bats (Fig. 3; the remaining 7 bats had lost their PIT tag; see Methods).

**Figure 4:**
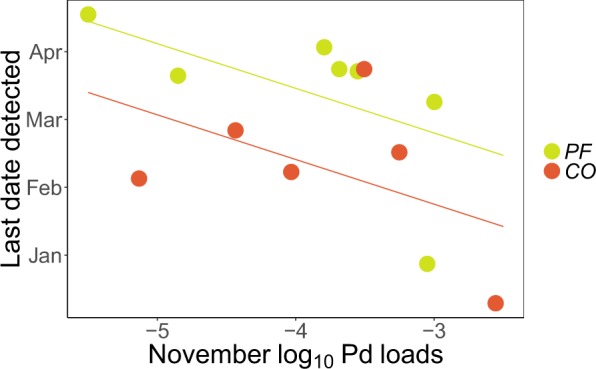
November *P*. *destructans* fungal loads (in nanograms) and the last date of detection for individual bats in the two treatment groups (Pf – *P*. *fluorescens*, Co – Control). *P*. *fluorescens*-treated bats were detected later than Control bats, and the last date of detection decreased (was earlier) with higher November *P*. *destructans* loads (control vs. *P*. *fluorescens* coef = 1.05 ± 0.58 months, t = −1.91, one-tailed P = 0.050, early winter fungal loads: coef = −0.66 ± 0.34 months/ng *P. destructans*, t = −1.93, one-tailed P = 0.041).

The last date a bat was detected on the PIT tag reader was over a month later for *P*. *fluorescens-*treated bats than control bats, and, overall, was earlier for bats with higher fungal loads in November (Fig. 4). We did not compare differences in fungal loads or UV-fluorescence among treatment groups in March for bats in the free-flying experiment because only three bats were found and recaptured when we visited the site on March 8. The remaining bats were likely in difficult-to-access portions of the mine.

### Cage experiment

**Figure 5:**
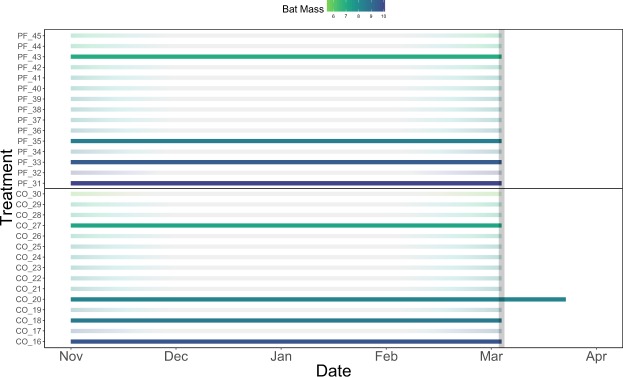
Survival, subsequent detection, and body mass of bats in the caged bat experiment. Each horizontal line represents a single bat, with line color indicating the body mass of that bat in November. Bats surviving until release March 8 (gray vertical bar) are shown by darker lines, and the single bat that was subsequently detected by the PIT tag reader is extended to the last date it was detected. Treatment is indicated by the first two letters of the bat identification number on the Y-axis (PF – *P*. *fluorescens*, CO – Control).

**Figure 6:**
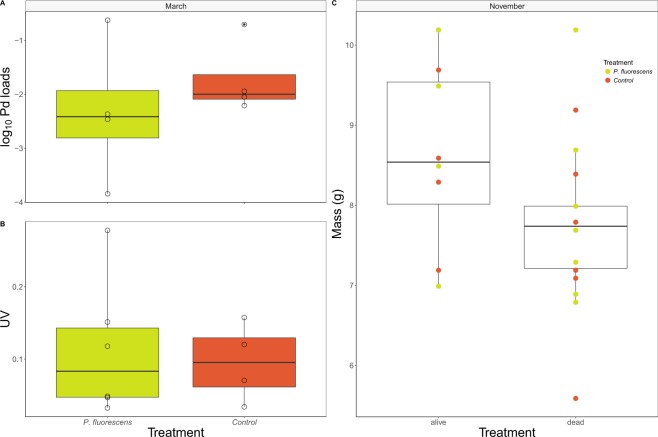
*P*. *destructans* (**A**) fungal loads, and (**B**) disease severity (UV) for bats in the cage experiment that survived to March 8, and (**C**) differences in November mass between bats in the cage experiment that survived to March 8, or died. (**A**) March fungal loads did not differ among treatment groups (treatment effect in model of log-transformed fungal loads: P = 0.77). (**B**) Disease severity (ultraviolet fluorescence) was not significantly different for *P*. *fluorescens*-treated bats than control bats (regression on arc-sin sqrt transformed data: control vs. *P*. *fluorescens*: coef = 0.017 ± 0.073, z = 0.234, P = 0.82). (**C**) Bats that were heavier in November had a higher probability of survival (logistic regression (reference group: control): Intercept: 20.1 ± 7.0; Body mass: 1.22 ± 0.44; P = 0.0054; *P*. *fluorescence* coef. −0.40 ± 0.97; P = 0.68).

On March 8^th^, 2016, four of 15 (26%) bats in the *P*. *fluorescens* cage and four of 15 (26%) bats in the control group cage were still alive; the others were dead (Fig. 5). Survival to March 8 was higher for bats with higher November body mass (which did not differ between treatment groups; Fig. 1) (Fig. 6C). Unlike in the free-flying experiment, *P*. *destructans* fungal loads in November were not a significant predictor of survival (P = 0.60). Most of the bats still alive in the cages were in very poor condition, and only one of the eight bats (from the control group) that survived to March 8 and was released was subsequently detected by the PIT tag reader (Fig. 5). Secondary measures of disease severity (Fig. 6A: fungal loads on bats in March and Fig. 6B: disease severity, as measured by UV-fluorescence) from the cage experiment showed non-significant differences among groups.

## Discussion

White-nose syndrome has caused widespread declines in multiple species of bats throughout eastern and central North America, with declines in *M*. *lucifugus* colonies in the first year of WNS detection averaging 79%^2,3^. Survival of untreated bats in the free-flying experiment was similarly severe, with 91% (95% CI: 62, 99%) of control bats likely dying over the winter. In the free-flying experiment, treatment with the probiotic, *P*. *fluorescens*, increased apparent overwinter survival more than five-fold by extending the last date of detection by a month into early spring. Although over half of *P*. *fluorescens*-treated bats still likely died from WNS over the winter, the effect of treatment was substantial.

Our results suggest that *P*. *fluorescens* could be a useful tool for reducing WNS impacts on bat populations, especially if treatment efficacy could be improved. One potentially important factor contributing to efficacy, based on work in other systems, is bacterial persistence and proliferation following treatment^23^. Increasing persistence and growth of *P*. *fluorescens* on bats by altering the dosage, or treatment frequency, or adding components to *P*. *fluorescens* solutions to encourage the formation of biofilms could help increase treatment efficacy, assuming these alterations would not have deleterious side effects^23^. In addition, previous *in vitro* studies suggested that many different strains of *P*. *fluorescens* have anti-*P*. *destructans* effects^16^. Future studies could examine alternate strains of *P*. *fluorescens* or other bacteria isolated from different populations (e.g., persisting populations of *M*. *lucifugus*^9^) or other species of bats (the strain in this study was isolated from *E*. *fuscus*;^16^).

The effect of *P*. *fluorescens* in reducing WNS impacts on other species has yet to be tested. The most important species to protect from WNS is *M*. *septentrionalis*, which suffers nearly 100% mortality, and is on a pathway toward extinction^2^. To date, no treatments have been developed for or tested on this species in either the lab or field. This is despite *M*. *septentrionalis* being the most heavily affected by WNS, with few hibernacula still containing this species in the US^2,7,24^.

Our results offer potential insights for the experimental design of field trials aimed at reducing WNS mortality in hibernating bats. Researchers often have to choose between cage-artifacts and concerns about bats leaving the site following treatment and uncertainty in the survival outcome for free-flying bats. Our data suggest that the free-flying experiment was a better experimental design than the cage experiment, despite some challenges. Bats in the free-flying experiment were able to roost and behave normally, and only one of 44 bats left the site on the day of the experiment, suggesting that the disturbance of handling and treatment are unlikely to compromise experiments if treatment can be done quickly (treatment, weighing and banding required ~65 minutes underground in this study). In addition, apparent survival was higher for bats with lower November fungal loads (i.e., loads were positively correlated with emergence date), as would be expected if bats were dying from WNS^24^, suggesting that WNS was the dominant source of mortality in the free-flying experiment. If bats were leaving the site due to disturbance, or simply to move between hibernacula, one would not expect a correlation between fungal loads and emergence date. The main challenge of the free-flying experiment was uncertainty associated with the fate of animals that were never detected by the PIT tag reader and not found dead within the site. However, as noted above, the extent of mortality in control bats inferred in the free-flying experiment was consistent with WNS declines observed in other populations of *M*. *lucifugus*. This supports the assumption that most bats that were not detected by the PIT tag reader after March 8 did not survive the winter. One final challenge with free-flying experiments is that treatment substances might be transferred among bats in different treatment groups (e.g., probiotic bacteria might be transferred from treated to control bats) and this could reduce the magnitude of treatment effects observed.

The cage experiment suffered from several shortcomings that, in hindsight, indicate this was a problematic design that should be modified if used in the future. In our experiment, we placed all the bats in each treatment into a single cage due to limited availability of space for mounting cages to natural substrate in a predator-protected room, and to allow social bats to roost in groups. This resulted in pseudo-replication in this experiment, as in most previous laboratory studies on WNS^1,17–19^. This is particularly problematic for studies of WNS, because in small cages bats appear to disturb other bats when they arouse from hibernation^20^, and increased arousal frequency is thought to be a key mechanism of WNS mortality^1,25,26^. The fact that survival in the cage experiment was correlated with initial body mass, but not fungal loads, suggests that disturbance from other bats, or an inability to move to other locations within hibernacula, was more important than WNS in determining survival in this experiment.

Together, these results indicate that the ideal design for a field trial (and for WNS challenge experiments more generally) is a free-flying experiment with mixed treatment groups in each site where bats have to pass through a PIT tag antenna to leave the site or are prevented from leaving the site (e.g. by sealing entrances, which may be very difficult). Sites where dead bats are relatively easy to find and are not eaten by predators (e.g. mice, rats, raccoons, or skunks) would reduce uncertainty in survival outcomes. Alternatively, if the site entrance(s) cannot be adequately covered with a PIT tag antenna, one should use replicated cages (constructed of metal, to prevent mice from chewing into the cages) with a small number of bats in each cage (one for bats that usually roost alone; a few for bats that roost in groups) to reduce cascading disturbances from infected bats. In addition, barriers to prevent larger predators (e.g. raccoons, skunks) from accessing the cages and eating the bats are an absolute necessity. Cages are not ideal in that they limit bats’ movement within sites, and individual bats within cages are pseudo-replicates (and thus need to be analyzed appropriately), but they offer higher certainty in terms of knowing the survival of each individual.

In conclusion, our results suggest that *P*. *fluorescens* could be a useful tool for reducing WNS impacts, and pairing *P*. *fluorescens* treatment with other approaches could stop species declines due to WNS^11^. Potential strategies that could be combined with treatment include reducing the environmental reservoir of *P*. *destructans*^3,27^, protecting and facilitating growth of populations of *M*. *lucifugus* that are now persisting with WNS, and improving summer and fall habitat for bats to increase reproductive success and fat storage for hibernation that would enable bats to better tolerate infection^21,28^. Increasing survival and reproduction could facilitate the evolution of resistance or tolerance which would eliminate the need for perpetual management action^29^. Finally, any strategy which slows or stops the very rapid local extirpations of *M*. *septentrionalis* colonies is urgently needed to prevent this species from extinction.

## Methods

We performed the field trial on *M*. *lucifugus* bats in the winter of 2015–16 at an inactive mine in southwest Wisconsin where *P*. *destructans* was detected the previous winter 2014–15. The mine has one large (~3 m tall by 5 m wide) entrance that was gated several years earlier, and a single smaller entrance that was sealed with a fine mesh metal screen the year of the gating. We selected a hibernacula where *P*. *destructans* had been detected the previous year because lab trials with *P*. *fluorescens* indicated that treating bats at the time of infection was more beneficial than treatment prior to infection^17^, and previous work suggests that most bats become infected early in the second year following *P*. *destructans* invasion, likely due to establishment of an environmental reservoir^3^. There were 226 *M*. *lucifugus* at the site in November 2014, before *P*. *destructans* was detected, but the colony had declined to 82 bats by March 2015. The average temperature where *M*. *lucifugus* roosted at this site was 7.0 °C ± 0.4 °C.

We screened 55 samples for *P*. *fluorescens* by PCR from bats collected in the winter of 2014–15 to confirm that *P*. *fluorescens* naturally occurred on bats found in the site to address concerns regarding using a live bacterium as a treatment. We found DNA from *P*. *fluorescens* or closely-related relatives present in 20% of samples and from all four species sampled (Little brown myotis (*Myotis lucifugus*), Northern long-eared myotis (*Myotis septentrionalis*), Tri-colored bat (*Perimyotis subflavus*), and Big brown bat (*Eptesicus fuscus*)).

In September 2015 we installed a PIT-tag reader (IS1001 and HPR reader, Biomark Inc., Boise, ID) at the site entrance (Fig. S1), and 3 metal screen cages (46 × 30 × 51 cm Fresh Air Screen Habitat, Zilla Products, Franklin, WI, USA) in a small chamber in the back of the mine where bats roosted in previous winters. We removed the top of each cage and mounted cages directly to the ceiling to allow bats direct access to the ceiling for roosting, and to allow for natural infection and reinfection. We also installed chicken wire with a hinged gate at the entrance of the cage room to prevent large predators (e.g. raccoons, skunks) from entering.

We briefly visited the site on Nov 16, 2015 (total time underground 14 minutes) to count the number of bats present and to assess the *P*. *destructans*-infection status of the bats at the site. We counted approximately 95 *M*. *lucifugus* and sampled six of them by dipping a sterile polyester swab in sterile water to moisten it and then rubbing the swab five times across both the forearm and muzzle of a bat^22^. We tested these samples for *P*. *destructans* DNA using qPCR^30^, and all six samples tested positive.

We returned to the site for the experimental treatment on Nov 20, 2015. We sealed off the entrance to the site (Fig. S1) using fine mesh cloth to prevent bats from leaving the site during the treatment. We collected all *M*. *lucifugus* we could find at the site (89 bats; 23 females and 66 males) and placed them individually in paper bags and brought them to a processing station near the entrance of the site. We weighed bats to the nearest 0.1 g with an electronic scale. We did not take a length measurement (e.g. forearm) to minimize handling time and disturbance. Recent work has shown that body mass is equally accurate in predicting fat stores (as measured by quantitative magnetic resonance) as body condition indices^31^. We sampled bats for *P*. *destructans* as described above and banded each bat with an aluminum band (2.9 mm; Porzana Ltd., Icklesham, E. Sussex, U.K.), that had a PIT tag attached (see Supplemental Methods text).

We randomly assigned bats to each of three treatment groups: control (N = 29 bats), *P*. *fluorescens* (31 bats), and a third treatment that will be reported elsewhere (29 bats). We treated each bat by spraying ~2 ml of a solution containing *P*. *fluorescens* (see Supplemental Methods text) on their wings and tail with a spray bottle (FantaSea, Blaine, WA). For control bats we replicated the handling disturbance but did not spray any liquid onto bats because our treatment could only be applied in liquid form and the goal of our study was to determine the effect of treatment compared to untreated bats. We split bats in each treatment group into the two experiments based on a power analysis (Fig. S2) – cage (15 for each treatment group in a single cage for each treatment; 5 females and 10 males per cage) and free-flying (16, and 14 bats in the *P*. *fluorescens* and control groups, respectively). After treatment, bats were released into cages or into the site onto a recovery cloth ~75 m away from the processing station. We removed the mesh from the site entrance so bats could freely pass through the opening surrounded by the PIT tag antenna (Fig. S1). We blocked off the rest of the entrance with screening to discourage bats from attempting to leave the site without passing by the PIT tag antenna. The total time underground was 65 minutes.

We returned to the site on March 8, 2016. We removed all the bats from the cages and captured all free-flying bats we could find (some portions of the site are inaccessible). Each bat was swabbed as described above, and one wing was photographed under ultraviolet (UV) light to measure an index of disease severity^32,33^. We then released all bats into the site. We downloaded data from the PIT tag reader on July 30, 2016 to determine the dates that bats with PIT tags were detected by the PIT tag reader. We note that detection by the PIT tag reader does not indicate the direction of travel when a bat is detected by the PIT tag reader (i.e. into or out of the mine). It only indicates that the bat was alive on that date and passed near (within ~15–20 cm) the PIT tag antennae.

When processing bats from the cage experiment, we noted that five of 16 (31%) of the PIT tags had become detached from the bands on the bats. As a result, we subsequently searched the site when no bats were present with a handheld PIT tag reader to determine whether free-flying had also lost their PIT tags. We found seven PIT tags that were not attached to bands, suggesting that known PIT tag loss in the free-flying group (seven of 24, or 29.2% of the bats that were never recorded on the PIT tag reader) was similar to that in the cage experiment (31%). There may have been additional bats in the free-flying group that lost their PIT tags (making our survival calculations underestimates), but there is no evidence that PIT tag loss differed by treatment group (cage and free-flying groups combined: *P*. *fluorescens* 4/31, control 4/29; Fisher’s exact test P = 0.93). We removed the bats who had lost their PIT tags (three *P*. *fluorescens*, one control) from analyses for the free-flying group since we could not detect them on the PIT tag reader. We added a new band with a PIT tag to any bat from the cage trial that had lost its PIT tag or band.

We determined the efficacy of the treatments by comparing apparent overwinter survival of bats in the treatments, with and without accounting for differences in initial individual fungal loads and body mass. We assumed bats that were never detected by the PIT tag reader died in the site because our PIT tag reader antennae provided full coverage of the entrance and had sufficient sensitivity to detect tags on flying bats. We assumed that any bats alive and detected by the PIT tag reader on or after March 8, 2016 had survived the winter (which we term “apparent overwinter survival”). We used March 8 as a cut-off for apparent overwinter survival because after March 8, 2016 surface temperatures near the mine were consistently above 2 °C (Fig. 1). Bats detected by the PIT tag reader prior to March 8, and never again, were assumed to have died or permanently emigrated. We searched all known sites within 50 km for banded bats and did not find any, suggesting that emigration to known sites was low.

We compared the latest date a bat was detected by the PIT tag reader between treatments as a continuous response variable of the last date a bat was known to be alive, while controlling for fungal loads and mass measured in November. Finally, we examined differences among treatment groups in UV fluorescence among individuals surviving the cage experiment, as an indicator of disease severity. We hypothesized that bats treated with *P*. *fluorescens* would have higher survival, have a later last detection by the PIT tag reader date, and lower UV fluorescence than the control group. We further hypothesized that apparent overwinter survival would increase with initial body mass and lower initial fungal loads in both experiments.

All research was performed as described under protocol Kilp1509 approved by the University of California, Santa Cruz’s IACUC committee. The work was exempt from FDA licensing, under regulation 21CFR 511.1.

## Supplementary information

Supplementary information

## Data Availability

The datasets generated during the current study are available from the corresponding authors on reasonable request.
