# Supplementary material for: Field trial of a probiotic bacteria to protect bats from white-nose syndrome
Source: Sci Rep. 2019 Jun 24;9:9158. doi: 10.1038/s41598-019-45453-z (PMC6591354; doi:10.1038/s41598-019-45453-z)
Supplement: Supplementary file 1 — Supplementary information [file 41598_2019_45453_MOESM1_ESM.pdf]

Field trial of a probiotic bacteria to protect bats from white-nose syndrome

Joseph R. Hoyt[1,2,*], Kate E. Langwig[2], J. Paul White[3], Heather M. Kaarakka[3], Jennifer A. Redell[3], Katy L. Parise[4,5], Winifred F. Frick[1,6], Jeffrey T. Foster[4,5], A. Marm Kilpatrick[1,*]

Supplemental Tables and Figures

Table S1. Progress on white-nose syndrome treatments. Bolded references are from published papers and an asterisk (*) indicates a conference presentation. Full abstracts, titles and author affiliations for the conference presentations can be found at www.whitenosesyndrome.org/wns-symposia-workshops.

**Treatment agent**	***In vitro***	**Lab Trial**	**Field Trial**
Chitosan	1*	2*	
Polyethelene glycol (PEG)	3*	4*	5*
Propolis	**6**		
Pseudomonas fluorescens	**7**	**8**	This study
Rhodococcus rhodocrous DAP 96253	**9**	10*	11*
Trichoderma sp.	12*		
Turbinafine	**13**	**14**	
Vaccine		**15**	16*
Valencia orange oil	**17**		

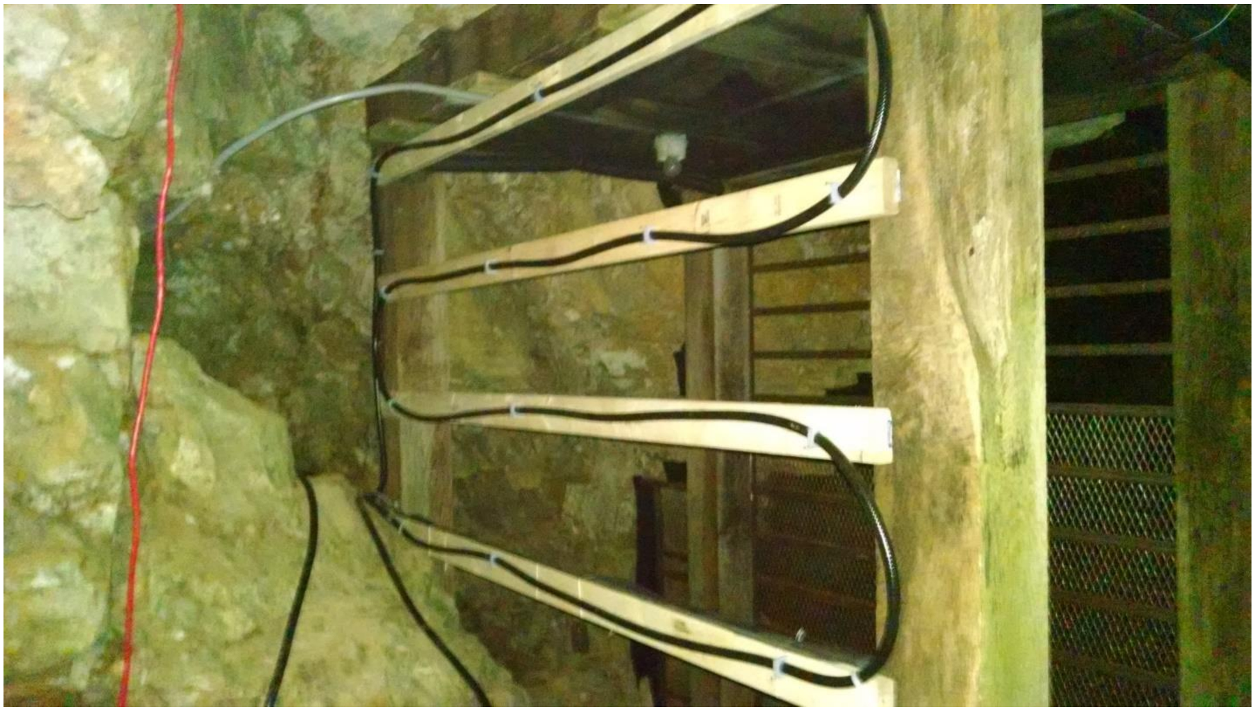

Figure S1. Photo of PIT tag antennae (black cable) installed at entrance of study site. The exit of the site is to the right. Shade cloth was used to prevent movement through the exit except between the boards with the attached PIT tag antenna.

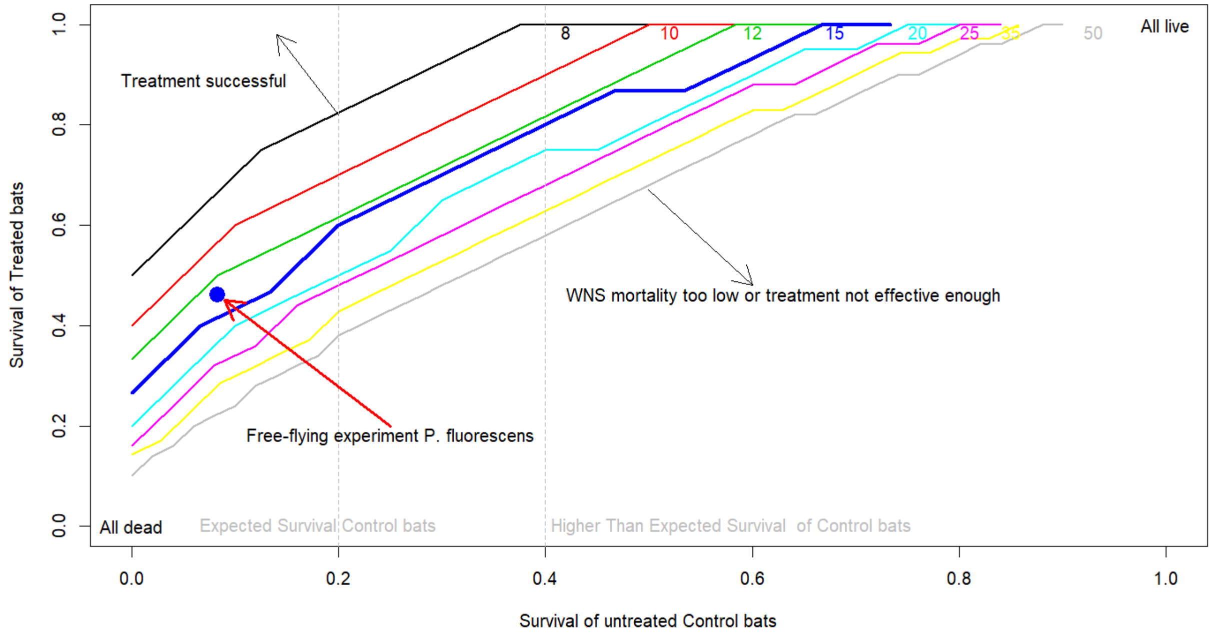

Figure S2. Power analysis used to design both experiments of the field trial. The x-axis shows survival of control (untreated bats), the y-axis shows survival of treated bats, and lines with different colors indicate sample sizes of bats in each treatment group that separate significantly different outcomes (above the line) from non-significant differences when results are analyzed with a fisher's exact test. The blue circle shows the outcome of the free-flying experiment which is just above the bold blue line which represents the sample size in this study, N = 15.

Supplemental Text
Methods
Measurement of fluorescence on bat wings under ultraviolet light
We took pictures of bats wings using a digital camera, approximately 15 cm above the wing under illumination with an UV light. We quantified the fraction of bat's wings (the area of the plagiopatagium proximal to the fifth digit, and below the radius) that fluoresced orange under ultra-violet light using Adobe photoshop, as the number of orange pixels divided by the total number of pixels in the photos of bats' wings.

PIT tag attachment to bands
We attached a PIT tag (12mm; Biomark Inc., Boise, ID) to the lip of each aluminum band using super glue (Loctite super glue gel control; Henkel corporation, Rock Hill, CT, USA). The lip of the band was abraded using 100 grit sand paper and the PIT tags were chemically etched using commercially available glass etching cream (Armour etch; Armour products, Hawthorne, NJ, USA) to provide maximum adhesion between the band and the PIT tag. We glued PIT tags to bands rather than gluing them directly to bat's backs to minimize disturbance and time underground (30-60 sec. per bat for glue to dry).

Preparation of treatment solution
The *P. fluorescens* treatment solution was prepared before the field trial by plating bacteria from frozen stock on sabouraud dextrose agar (SDA). Colonies were allowed to grow for one day at room temperature then suspended in a 10X phosphate buffer (PBS) and glycerol solution, by flooding the plate. The solution was homogenized and serial dilutions were performed using an aliquot of the prepared solution under the same culturing conditions and the remaining liquid was frozen at 20°C. After determining the concentration from the serial dilution plates using colony-forming units (CFU), the remaining frozen liquid was diluted to $1x10^8$ CFU's. The bacterial solution was shipped overnight on ice and was applied to bats the following day to minimize CFU loss.

Literature Cited

1 Vonhof, M. J., Carter, T. C., Keel, M. K. & Reeder, D. M. Test of a biocompatibile, biodegradable, widely available and inexpensive anti-fungal agent on the growth of *G. destructans*, the causative agent of White-nose Syndrome, on experimentally-infected bats under controlled laboratory conditions. *White-nose syndrome workshop* (US FWS, Madison, WI, 2012).
2 Vonhof, M. J., Carter, T. C., Eversole, R. R. & Keel, M. K. Testing the Efficacy of Chitosan to combat growth of *Pseudogymnoascus destructans* on Experimentally-Infected Little Brown Bats. *White-nose syndrome workshop* (US FWS, Grand Rapids, MI, 2015).
3 Perryman, J., Turner, G. & Overton, B. Evaluation of *Epicoccum nigrum* and Polyethylene Glycol (PEG 8000) in the control of *Pseudogymnascus destructans* (Blehert & Gargas) Minnis & D.L. Lindner. *White-nose syndrome workshop* (US FWS, 2014).
4 Willis, C. K. R. To spray or not to spray: Experiments and models to evaluate alternative management approaches for white-nose syndrome. *White-nose syndrome workshop* (US FWS, Nashville, TN, 2017).

5 Turner, G., Overton, B., White, J. P., Scafini, P. M. & Lilley, J. B. T. Field Application of Polyethylene Glycol 8000 as a Control for White-nose Syndrome in Naïve Bats. *White-nose workshop* (USFWS, 2016).

6 Ghosh, S. *et al.* Evidence for Anti-Pseudogymnoascus destructans (Pd) Activity of Propolis. *Antibiotics* **7**, 2 (2017).

7 Hoyt, J. R. *et al.* Bacteria isolated from bats inhibit the growth of *Pseudogymnoascus destructans*, the causative agent of white-nose syndrome. *PLoS One* **10**, e0121329, doi:DOI: 10.1371/journal.pone.0121329 (2015).

8 Cheng, T. L. *et al.* Efficacy of a probiotic bacterium to treat bats affected by the disease white-nose syndrome. *J. Appl. Ecol.* **54**, 701-708, doi:10.1111/1365-2664.12757 (2017).

9 Cornelison, C. T. *et al.* A preliminary report on the contact-independent antagonism of *Pseudogymnoascus destructans* by *Rhodococcus rhodochrous* strain DAP96253. *BMC Microbiology* **14**, 246, doi:10.1186/s12866-014-0246-y (2014).

10 Amelon, S. K., Cornelison, C. T., Hooper, S. & Lindner, D. In vivo evaluation of fungistatic volatile compounds from native soil bacteria *Rhodococcus rhodochrous* DAP96253 for inhibition of *Pseudogymnoascus destructans* in bats. *White-nose syndrome workshop* (US FWS, St. Louis, MO, 2014).

11 Amelon, S. K., Cornelison, C. T., Hooper, S. & Lindner, D. in *White-nose syndrome workshop.* (US FWS).

12 Zhang, T., Chaturvedi, V. & Chaturvedi, S. Trichoderma polysporum-A Biocontrol Agent. *White-nose syndrome workshop* (US FWS, Grand Rapids, MI, 2015).

13 Souza, M. J., Cairns, T., Yarbrogh, J. & Cox, S. In vitro investigation of a terbinafine impregnated subcutaneous implant for veterinary use. *Journal of drug delivery* **2012** (2012).

14 Court, M. H. *et al.* Pharmacokinetics of terbinafine in little brown myotis (*Myotis lucifugus*) infected with *Pseudogymnoascus destructans*. *Am. J. Vet. Res.* **78**, 90-99 (2017).

15 Rocke, T. E. *et al.* Virally-vectored vaccine candidates against white-nose syndrome induce anti-fungal immune response in little brown bats (*Myotis lucifugus*). *Scientific Reports* **9**, 6788 (2019).

16 Rocke, T. Development and testing of vaccine candidates against white nose syndrome in bats *White-nose syndrome workshop* (US FWS, Nashville, TN, 2017).

17 Boire, N. *et al.* Potent Inhibition of *Pseudogymnoascus destructans*, the Causative Agent of White-Nose Syndrome in Bats, by Cold-Pressed, Terpeneless, Valencia Orange Oil. *Plos One* **11**, e0148473, doi:10.1371/journal.pone.0148473 (2016).
